# The bridge of the gut–joint axis: Gut microbial metabolites in rheumatoid arthritis

**DOI:** 10.3389/fimmu.2022.1007610

**Published:** 2022-10-06

**Authors:** Xiaoyu Xu, Miao Wang, Zikang Wang, Qian Chen, Xixuan Chen, Yingyue Xu, Min Dai, Bin Wu, Yanping Li

**Affiliations:** ^1^ College of Traditional Chinese Medicine, Chongqing Medical University, Chongqing, China; ^2^ Department of Rheumatology, Chongqing Hospital of Traditional Chinese Medicine, Chongqing, China

**Keywords:** rheumatoid arthritis, gut microbial metabolites, intestinal barrier, immune balance, bone destruction

## Abstract

Rheumatoid arthritis (RA) is an autoimmune disease characterized by joint destruction, synovitis, and pannus formation. Gut microbiota dysbiosis may exert direct pathogenic effects on gut homeostasis. It may trigger the host’s innate immune system and activate the “gut–joint axis”, which exacerbates the RA. However, although the importance of the gut microbiota in the development and progression of RA is widely recognized, the mechanisms regulating the interactions between the gut microbiota and the host immune system remain incompletely defined. In this review, we discuss the role of gut microbiota-derived biological mediators, such as short-chain fatty acids, bile acids, and tryptophan metabolites, in maintaining intestinal barrier integrity, immune balance and bone destruction in RA patients as the bridge of the gut–joint axis.

## Introduction

The results of a large number of animal model studies of arthritis and preclinical studies conducted at an early stage of onset (the latency period) suggest that alterations in gut microbial composition accelerate the onset of arthritis-associated diseases (1, 2). Intestinal dysbiosis can initiate and persist in the inflammatory response of the intestinal mucosa, which can lead to dysfunction of the intestinal epithelial barrier by disrupting the tight junctions between intestinal epithelial cells and increasing intestinal permeability. This phenomenon is called “intestinal leakage” (3). As a result of severe bacterial migration, the re-circulation of aberrantly activated immune cells from intestinal sites to secondary lymphoid organs and arthritic joints is a possible mechanistic link between mucosal alterations and arthritis development (4–6). Rheumatoid arthritis (RA) is an autoimmune disease characterized by chronic inflammation of the joints (7, 8). A recent study published in Nature Communications found increased serum zonulin expression and decreased intestinal tight junction protein expression in patients with new-onset RA. In mouse models of arthritis, intestinal inflammation occurred earlier than the onset of arthritis, and targeting the intestinal barrier or zonulin alleviated arthritis. The study suggested that targeting the intestinal barrier is a potential strategy for treating autoimmune diseases such as RA (9).

Some scholars have proposed that intestinal ecological disorders are related to the autoimmune mechanism involved in the development of RA (10). The gut microbiota produces metabolites that regulate host immunity and influence host resistance and susceptibility to disease. It plays a crucial role in maintaining intestinal barrier function. A 2013 Nature publication demonstrated that metabolites from gut microbes mediate communication between the commensal microbiota and the immune system, affecting the balance between the mechanisms for the generation of anti-inflammatory regulatory T (Treg) cells or proinflammatory T helper 17 (Th17) cells (11). Recent publications in Cell Host & Microbe showed that gut microbes respond to host immune activation by rapidly altering gene transcription and immunomodulatory metabolites before changes in community composition are detected (12). New studies have found that RA patients have metabolic disorders associated with lipids, amino acids, carbohydrates, and the metabolites of gut microbes that are involved in the above metabolic pathways. Among them, gut microbial metabolites, such as short-chain fatty acids, bile acids (BAs), tryptophan, and their metabolites, have immunomodulatory effects on different subtypes of immune system and nonimmune cells, including the intestinal epithelium (13). Therefore, we speculate that the interactions of the metabolites of intestinal microorganisms and immune regulation is the key to building the gut–joint axis bridge.

In recent years, it has been indicated that gut microbiota dysbiosis is associated with the development of multiple chronic inflammatory joint diseases, including RA (14–18). Glucocorticoid-induced loss of beneficial gut bacterial extracellular vesicles is associated with the pathogenesis of osteonecrosis. Beneficial gut bacterial extracellular vesicles can enter the femoral head, increase angiogenesis and bone formation, reduce apoptosis, and alleviate bone destruction symptoms (19). Jones et al. (20). proposed the concept of “Osteomicrobiology” and said that intestinal epithelial permeability can affect the rate of bone turnover (turnover), while the microflora can regulate bone development, bone ageing, and pathological bone loss after birth.

However, the mechanism of action of the gut–joint axis remains incompletely understood. Therefore, we speculate that the metabolites of gut microbes can serve as a bridge to the gut–joint axis in RA, play a key role in regulating the intestinal barrier and immune balance, and participate in bone metabolism; this hypothesis may also provide insights into the aetiological mechanisms of RA (9, 21, 22). Therefore, we review gut microbiota-derived metabolites in the context of RA pathogenesis and the gut–joint axis, discussing their role in the maintenance of the gut barrier, immune balance, and bone destruction. This review will provide a theoretical basis for further research on novel pharmacological targets and for applying dietary and pharmaceutical interventions to prevent and treat diseases.

## Role of bile acids in the gut–joint axis

Elevated levels of primary BAs have been found in the faeces of some RA patients, indicating their potential to predict RA arthritis severity (23–25). Increased bile acid in RA patients andmice has been shown to reduce the number of swollen andtender joints, erythrocyte sedimentation rate (ESR) (26), Creactiveprotein (CRP) (27), anti-CCP antibodies RF-IgM, RFIgGand RF-IgA (28). Primary BAs, such as cholic acid (CA) and chenodeoxycholic acid (CDCA), are synthesized from cholesterol in the liver and can be accomplished by two different pathways (29). The classic pathway involves the transfer of cholesterol via cholesterol-7 α-hydroxylase (CYP7A1), which catalyzes the generation of 7 a-hydroxycholesterol, which further catalyzes the generation of CA and CDCA (30, 31). Under normal conditions, at least 75% of BAs are produced through this pathway. The alternative (or acidic) pathway is catalyzed by sterol-27-hydroxylase (CYP27A1), producing 27-hydroxycholesterol, which is further acted upon by 25-hydroxycholesterol 7-α-hydroxylase (Cyp7b1) to generate CDCA (32). The gut microbiota regulates the expression of the enzymes CYP7A1, CYP7B1, and CYP27A1 (33). In addition, approximately 5%-10% of BAs are secreted into the colon and mainly biotransformed by the gut microbiota to secondary BAs (SBAs) or excreted *via* faeces as BAs (34). In turn, the SBAs may regulate the synthesis of BAs through negative feedback. The biotransformation of BAs performed by the gut microbiota includes deconjugation, oxidation, and epimerization of 3-, 7-, and 12-hydroxyl groups; 7-dehydroxylation; esterification; and desulfation (35–37). Bile acid deconjugation is carried out by bacteria with bile salt hydrolase (BSH) activity, mainly including Lactobacillus, bifidobacterium, Clostridium and bacteroids (38, 39). These SBAs that are metabolically produced by gut microbes participate in the inflammatory response of the gut (40, 41). Several recent studies have identified immunomodulatory properties of BAs in the gut (42–44).These microbiota-modified BAs play an essential role in the pathogenesis of RA; these functions involve mucosal barrier homeostasis, immunomodulation, and improving bone erosion.

### Bile acids regulate intestinal barrier of RA

The gut mucosal barrier is a “natural guard” in the human body, preventing pathogens from invading the intestine into the systemic circulation and extra-intestinal tissues. Alterations in gut microbiota (dysbiosis) can lead to many autoimmune diseases, notably RA. The previous study demonstrated that the intestinal barrier was disrupted in patients with RA, accompanied by dysbiosis and inflammatory activation of the gut (9). A complex, interactive network of cells and cytokines has been proven to be involved in the pathogenesis of RA. TNF‐α and IL‐6 play a central role in the pathogenesis of RA. Recent studies have suggested that additional proinflammatory cytokines, such as IL-7, IL-17, IL-21, IL -23, GM-CSF, IL-1β, IL-18, IL-33 and IL-2 are also involved in the pathogenesis of RA (45, 46). These cytokines can cause a proinflammatory response and exacerbate intestinal epithelial barrier damage (47). Secondary BAs such as deoxycholic acid (DCA) and lithocholic acid (LCA) can suppress macrophage TNF-α production *via* FXR (30, 48). Renga et al. discovered that FXR/mice (FXR deficient) had a higher incidence of colonic inflammation than wild-type mice (49). Another research revealed that the accumulation of LCA and DCA in the liver promoted the expression of tight junction protein and blocking protein while reducing the secretion of inflammatory cytokines (TNF-α and IL-6) to protect the intestinal barrier (50). Furthermore, some DCA and LCA derivatives, such as HDCA, UDCA, and isoLCA, were reported to be strongly negatively connected with proinflammatory cytokines (IL-6, TNF-α, and IL-1β) levels and positively correlated with colon length and mucin two levels *via* bile acid nuclear receptors (51). Guo et al. (52). demonstrated that secondary BAs, especially LCA, can inhibit IL-1β production in macrophages. These cytokines are sufficient to promote a state of chronic mucosal inflammation, thereby disrupting intestinal homeostasis and destroying the mucosa (53), increased intestinal epithelial cell death (54), disrupt the intestinal epithelial tight junctions (55).In another study, LCA negatively regulates NLRP3 inflammasome activation through the G‐protein‐coupled receptor‐5 (TGR5)‐cAMP‐PKA axis to ameliorate intestinal inflammation (56).In recent years, progress in research on the pathogenesis of RA has resulted in the development of new anti-rheumatic drugs, such as biological agents and small-molecule targeted signalling pathway inhibitors (57).However, clinical trials of approved biological agents for RA, including TNF-α inhibitors, IL-6 receptor inhibitors, T cell co-stimulation blockers, B cell eliminators, and intracellular signal inhibitors of the JAK pathway showed only less than 50% of RA patients could benefit from these new drugs (58). Furthermore, these treatments are usually associated with adverse reactions, such as cardiovascular and gastrointestinal bleeding risk, liver and kidney toxicity, growth inhibition, infection, and risk of tumours (59). Bile acids are to augment gut barrier function and anti-inflammatory effects through multiple mechanisms. Its functional role and potential therapeutic utility in RA are worth further exploration (60). Bile acids expressed by ISCs can be also be responsible for gut epithelium’s homeostasis and regeneration, which is required for intestinal epithelial homeostasis and protection from DSS-induced colitis (61). LCA and DCA promote intestinal organoid regeneration by activating TGR5 in intestinal stem cells (ISCs) through a proto-oncogene tyrosine protein kinase (SRC)/Yes associated protein 1 (Yap-mediated regeneration mechanism (62). It can protect the intestinal mucosal barrier’s function and maintain the joint and intestine balance.

### Regulation of immune balance by bile acids

BAs act as signalling molecules that regulate immune homeostasis, and BA metabolites control the mechanism underlying host immune responses by directly regulating the balance of Th17 and Treg cells. Restoration of the intestinal BA pool increases colonic ROR γ + Treg cell counts and improves host susceptibility to inflammatory colitis *via* nuclear BA receptors (43). Thus, the pangenomic biliary network interactions between the host and its bacterial symbionts may control host immune homeostasis through the metabolites produced (43). Transplantation of the microbiota from mice susceptible to collagen-induced arthritis (CIA) into germ-free mice increased the severity of arthritis, serum interleukin (IL)-17 concentrations, and the proportion of CD8+ and IL-17 (Th17)-producing T cells (63). It is well known that immune system disorders are mainly caused by an imbalance between Th17 and Treg cells. Clinical studies have found that the percentage of Treg (CD4 + CD25high CD127 -) cells is lower in RA patients than in osteoarthritis patients or healthy controls. The proportion of Th17 (CD4 + CCR6 + CXCR3 -) cells is higher in RA patients than in healthy controls (64). Peripheral T (preg) cells were demonstrated to suppress immune responses during colonization and support metabolic functions of the gut microbiota. Campbell et al. (44) found that the SBA 3 β-hydroxydeoxycholic acid (isoDCA) is a potent inducer of preg cells. Colonization with an isoDCA-producing microbial community can promote the generation of colonic preg cells in a cns1-dependent manner. In the BA metabolite pools, 3-oxoLCA and isoalloLCA are two different derivatives of LCA that can both act as T-cell regulators in mice. Administration of 3-oxoLCA and isoalloLCA to mice reduces Th17-cell differentiation in the intestinal lamina propria and increases Treg cell differentiation (65). The study showed that 3-oxoLCA acts by directly binding to the key transcription factor retinoid-related orphan receptor-γ t (ROR γ t) to inhibit the differentiation of Th17 cells and that isoalloLCA increases Treg cell differentiation *via* cns1 (65). Xiao et al. (66) synthesized the sulfated BA metabolite LCA-3-s and demonstrated that LCA-3-s functions by targeting ROR γ T to selectively inhibit Th17-cell differentiation without affecting Th1, Th2, and Treg cells. It was shown that disorder of BA metabolism could lead to decreased levels of sulfated BA metabolites and disrupt the Th17/Treg immune balance (66). These data indicate that the levels of these BAs are negatively correlated with the expression of Th17-cell-related genes and are involved in regulating the Th17/Treg immune balance.

Together, the gut microbiota and its SBA metabolites interact with the host and can help maintain gut homeostasis through different BA receptors and cell signalling pathways. The above studies confirmed the findings in mouse models and improved our understanding of the relationship between BAs and their microbial producers and intestinal inflammation. Interestingly, the immunomodulatory properties of newly discovered SBA derivatives reveal the potential value of BAs as cellular signalling factors. However, most studies have focused on the effects of SBA derivatives on the differentiation of Treg and Th17 cells, whereas other T-cell subtypes have been less well-studied. BAs holds great potential for application in clinical prognostication and therapy in the context of the gut microbiota-articular axis. However, further research should focus on deepening our knowledge of the mechanistic links and translating them into clinical practice.

### Effects of bile acids on bone destruction

Recent studies have found that BAs are closely related to bone metabolism. Vitamin D levels in blood circulation are positively correlated with BA levels (67). Vitamin D is a fat-soluble vitamin that plays an essential role in calcium regulation and bone health (68). BAs can promote intestinal absorption of fat-soluble vitamin D, improve bone density, reduce fracture rates, and improve bone destruction in patients (69–71). Among them, the receptors of SBA metabolites are expressed in immune cells. The close interaction between these immune cells and bone cells regulates bone health and influences bone metabolism and density (72). Studies have proven that proinflammatory cytokines such as TNF-α, IL-1β, and IL-6 can inhibit the osteogenic differentiation of stem cells (73). For example, TNF-α accelerates the expression of P2Y2 receptors and inhibits Wnt/β-Catenin signalling to inhibit cell proliferation and osteogenic differentiation of mesenchymal stem cells (MSCs) (74, 75). IL-1β and IL-6 also reduce cell proliferation and osteogenic differentiation of bone marrow stromal cells (BMSCs) (73, 76–78). Ursodeoxycholic acid inhibits the production of proinflammatory cytokines, such as TNF-α, IL-1β, and IL-6, reducing inflammation-related side effects to improve destructive bone lesions (79). In addition, BAs affect bone metabolism and bone density *via* G protein-coupled BA receptor 5 signalling pathways by activating FXR (80). Numerous studies have shown that the BA receptor FXR can inhibit osteoclastogenesis and promote osteoblast formation (81, 82). A previous study showed that the osteoclast count, osteoclast surface, and bone density were significantly reduced in 8- to 20-week-old FXR −/− mice compared with FXR + mice (83). CDCA, a BA breakdown product, can activate FXR, increase alkaline phosphatase activity and extracellular matrix calcification, and stimulate the expression of osteoblast marker genes (bone sialoprotein (BSP), osteocalcin (OC), osteopontin (OPN) and alkaline phosphatase (ALP)) and the DNA-binding activity of the bone transcription factor Runx2 (84). In conclusion, BAs alleviate bone destruction and patient symptoms.

## Role of short-chain fatty acids in the gut–joint axis

Most of the breakdown, emulsification, and absorption of nutrients occurs in the small intestine. However, the human body lacks the enzymes necessary to digest some complex carbohydrates, such as those present in dietary fibre. However, gut microbes can help metabolize these nondigestible carbohydrates to different SCFA molecules (e.g., acetate, butyrate, and propionate) (85–88). These SCFA molecules can regulate multiple metabolic pathways both in the gut and outside the gut and are associated with a variety of physiological processes, such as energy balance, sugar/lipid metabolism, inflammation, and even immune regulation (89–91). In addition, some SCFAs also play a role in regulating the intestinal barrier. For example, propionate and butyrate have repeatedly been described as energy sources essential for colonocyte proliferation and maintenance of the intestinal barrier (92). Unlike propionate and butyrate, succinate is often ignored, and currently, the effect of succinate on inflammation remains controversial (93–97). It has been confirmed that RA is associated with a decrease in the abundance of bacteria that produce SCFAs, mainly propionate and butyrate (98). For example, 51 (92.7%) of the 55 RA-associated strains were butyrate-consuming bacteria, 46 of which were enriched in patients with RA compared with only five enriched in healthy controls, suggesting that patients with RA exhibited more significant enrichment of butyrate-consuming flora. Adjustment of the gut microbiota to increase the abundance of butyrate-producing species and reduce butyrate consumption can lead to increases butyrate production, improve the inflammation status and lead to a good prognosis (98). Another study showed that the mean levels of acetate, propionate, butyrate, and valerate decreased in RA patients, as shown by analysis of clinical samples, but in mice, administration of the first three SCFAs improved arthritis symptoms before the mice developed CIA (99).

In conclusion, SCFAs involved in the RA gut–joint axis may play a role in protecting against RA by protecting the integrity of the intestinal environment, enhancing barrier function, and regulating immune cells to improve inflammatory responses and bone metabolism.

### Short-chain fatty acids regulate the intestinal barrier

The relationship between SCFAs and the intestinal barrier may have significant implications in the pathogenesis and treatment of RA. According to the gut–joint axis hypothesis, the main mechanism of intestinal barrier disruption in RA is increased production of zonulin. Zonulin is a master regulator of tight junction integrity in intestinal epithelial cells (100) and can cause the breakdown of the proteins zo1 and occludin from the tight junction complex (101), leading to intestinal barrier damage, increased vascular barrier permeability, translocation of bacterial products in the blood, and initiation of inflammatory responses.

According to the gut–joint axis hypothesis, the main mechanism of gut barrier disruption in RA is increased zonulin production, and zonulin also appears to be a potential therapeutic target (100). In mice with CIA, which is a type of inflammatory arthritis characterized by impaired gut permeability and gut microbial dysbiosis, dietary supplementation with butyric acid decreased serum zonulin concentrations and upregulated the expression of tight junction proteins, thereby restoring intestinal permeability (102). Interestingly, targeted therapy with zonulin (indirectly with butyrate and directly with pyrazole acetate) prevented arthritis attacks and reduced arthritis symptoms, supporting the gut–joint axis hypothesis. Butyrate may promote intestinal barrier function by upregulating the tight junction protein (ZO-1) promoter. Treatment of human clonal colon adenocarcinoma (Caco-2) cells with sodium butyrate resulted in decreased permeability and increased expression of ZO-1 and occludin. This effect was attenuated by the treatment of Caco-2 cells with GPR109A-interfering shRNA (103), demonstrating that the effect was probably produced by butyrate acting on GPR109A. The claudin family of proteins is also involved in the composition of tight junctions, and in IEC cells 37, butyrate was also found to promote claudin-1 expression (104).

Interestingly, SCFAs were shown to be able to inhibit LPS-induced NLRP3 inflammasome activation, which is a consequence of inhibition of the HDAC pathway. NLRP3 inflammasome activation can have deleterious effects on intestinal barrier integrity (105). One study found significant overexpression of NLRP3 in the inflamed gut of SKG mice, and treatment of SKG mice with mcc950, an NLRP3 antagonist, prior to induction of inflammatory disease inhibited the development of intestinal disease and delayed the onset of joint inflammation (106). In addition, several clinical studies have shown that activation of NLRP3 is associated with systemic or local inflammation in RA, and *in vivo* studies have also shown enhanced expression of NLRP3 inflammasome components in macrophages and that NLRP3 deficiency suppresses the development of arthritis and cartilage destruction. In animal experiments, the expression of NLRP3, caspase-1, and IL-1 was increased in the serum and knee synovium of mice with CIA. Mucoprotein (MUC) expression affects bacterial adhesion, promotes the adhesion of the beneficial species *Lactobacillus acidophilus* and *Bifidobacterium longum* to intestinal epithelial cells, and inhibits the adhesion of *Escherichia coli* (107). SCFAs can enhance MUC expression in intestinal secretory goblet LS174T cells by inhibiting HDACs and subsequent histone acetylation and methylation and activating AP-1 (108). Jung et al. (107) further demonstrated that incubation of LS174T cells with butyrate solution increased the expression of MUC3, MUC4, and MUC12 but had no effect on MUC2 expression. In addition to mucins, intestinal epithelial cells produce a group of peptides with direct antimicrobial activity, such as lectins, defensins, and cathelicidins, which play an antimicrobial role to regulate microbial populations, preventing bacterial overgrowth and dysbiosis, and play an important role in host intestinal barrier defence against microorganisms. *In vivo* studies have shown that SCFAs, especially butyrate, enhance the expression of LL-37, the only member of the cathelicidin family, in human colonocytes. In contrast, the interactions of SCFAs with their receptor gpr43 promote the expression of C-type lectin domains in mouse and human intestinal epithelial cell cultures and the expression of defensins 1, 3, and 4 (109, 110). In addition, SCFAs can also alleviate colonic inflammation by inducing IL-22 production and maintaining intestinal barrier integrity and immune balance in mice through interaction with the gpr43 receptor (111), thereby alleviating colonic inflammation (112).

### Regulation of immune balance by short-chain fatty acids

SCFAs can achieve direct anti-inflammatory effects through different pathways, but these effects are all achieved through inhibition of the transcription factor NF-κB, such as by inhibition of the histone deacetylase (HDAC) pathway and activation of the peroxisome proliferator activated receptor γ (peroxisome proliferator-activated receptor γ) pathway, which can both inhibit downstream NF-κB (113, 114). NF-κB can downregulate the expression of several mediators that contribute to the inflammatory response, such as proinflammatory cytokines, chemokines, iNOS and COX-2 enzymes, adhesion molecules, growth factors, acute phase proteins, and immune receptors (115).

Macrophages are essential in the pathogenesis of RA. An increase in the number of macrophages in the synovium is an early marker of active rheumatic disease, and large numbers of macrophages are a prominent feature of inflammatory lesions. The extent of synovial macrophage infiltration is correlated with the extent of joint erosion, and removal of these macrophages from inflamed tissue has profound therapeutic benefits. Butyrate downregulates lipopolysaccharide-induced proinflammatory mediators, including NO, IL-6, and IL-12, after stimulation of murine bone marrow-derived and intestinal lamina propria macrophages with LPS and butyrate (116). Butyrate was shown to significantly increase the clearance of intracellular Salmonella cells as early as 30 min after macrophage infection and to maintain this state for three hours (117). Schulthess et al.found that butyrate exposure during macrophage differentiation enhanced antibacterial activity and reduced pathogen dissemination by inhibiting HDACs. We demonstrated that butyrate has immunomodulatory effects on macrophages.

Dendritic cells (DCs) are critical antigen-presenting cells linking the innate and adaptive immune responses, inducing the priming and differentiation of naïve CD4 + T cells and cross-priming of CD8 + T cells and promoting B-cell antibody responses. Therefore, DCs also play a key role in maintaining immune homeostasis and toleranceDC-T-cell interactions underlie the generation of autoimmune responses in RA. DCs are found in the synovium and joint fluid in RA, often in the centre of T-cell clusters. These DCs express MHC II, the costimulatory molecules CD40, CD80, and CD86, adhesion molecules such as DC-SIGN, and chemokine receptors such as CCR7, as described in early manuscripts (Ref.). Moreover, DCs in the RA synovium secrete chemokines that attract proinflammatory immune cells, including macrophages, neutrophils, and monocytes (118). DCs can polarize T cells to a Th1 or Th2 phenotype depending on the cytokine environment and participate in the development and progression of RA. SCFAs similarly regulate DC function. Among them, propionate and butyrate can block the differentiation of murine myeloid progenitors to DCs. Upon entry into dendritic progenitor cells *via* the transporter scl5a8, SCFAs act as inhibitors of HDAC1 and HDAC3 isoforms, thereby inhibiting pu1 and RelB, two transcription factors critical for the differentiation of DCs (119). Butyrate-treated RA DCs showed decreased CD11c (CD11c is an important DC biomarker) but increased CD103 (a biomarker for mucosal DCs in mesenteric lymph nodes and parts of the lamina propria of the colon) and integrin α four β 7 (a β integrin expressed on mucosal lymphocytes, NK cells and eosinophils involved in lymphocyte homing to Peyer’s patches and lamina propria in the gut) levels. In addition, butyrate significantly inhibited the CD4+ T priming capacity and retinal dehydrogenase levels of RA DCs (120).

In addition, butyrate can also act directly on T cells to promote extrathymic Treg cell generation in the absence of DCs. Butyrate also increases the levels of Treg cells in the colon by inhibiting HDAC activity at the FOXP3 locus. Butyrate treatment ameliorated TNBS-induced colitis in mice because butyrate increased the number of CD25 Foxp3 Tregs in peripheral blood and the colon, increased IL-10 and IL-12 levels in peripheral blood and the colon, and decreased IL-17 and IL-23 levels in mesenteric lymph nodes (121). The underlying mechanism may the targeting of CD103 DCs by SCFA such as butyrate, which have gut homing functions, contribute to intestinal T-cell differentiation, and induce tolerogenic effects in the intestinal mucosa by promoting the differentiation of Foxp3 Treg cells (122). Further mechanistic studies showed that activation of GPR109A by administration of SCFAs to mice increased retinal aldehyde dehydrogenase-2 (Raldh2) expression, promoted the tolerogenic activity of CD103 DCs in mesenteric lymph nodes, and promoted Treg differentiation byccatalyzingthe conversion of vitamin A to retinoic acid *via* Raldh2 (123). In a study by Chen et al. (124), butyrate promoted Th1 levels but induced the expression of T box transcription factor (T-bet) and the anti-inflammatory cytokine IL-10 by inhibiting HDAC activity independently of gpr43 activation, thereby suppressing Th17 polarization of naïve T cells in culture and *in vivo*. In RA patients, higher butyrate levels were associated with increased Treg levels (123). In a trial conducted in mice with CIA, SCFAs reduced the severity of arthritis by promoting the local and systemic expansion of circulating Treg cells, regulating IL-10 expression (125). In RA model mice, butyrate significantly inhibited the expression of IL-1β, IL-6 and IL-17A, which promoted the expression of IL-10. IL-10, thereby affecting the function of Th17 cells, increasing the systemic levels of Treg cells and decreasing those of Th17 cells (126).

In addition to being enhanced by butyrate, Treg cell generation in the periphery can be enhanced by propionate, another microbially derived SCFA that inhibits histone deacetylases (HDACs), but not acetate, which lacks this HDAC inhibitory activity (121). In clinical studies, patients with multiple sclerosis (MS) were administered propanoic acid (PA) as a supplementary immunotherapy, and after two weeks of PA intake, a significant and sustained increase in the levels of functional Treg cells was observed, whereas the levels of Th1 and Th17 cells decreased significantly. A *post hoc* analysis showed that (127) intake of PA for three years resulted in lower annual relapse rates, stabilization of disability, and reduced brain atrophy. Functional microbiome analysis revealed increased expression of Treg cell-inducible genes in the gut after the intake of PA.

Furthermore, PA normalizes Treg cell mitochondrial function and morphology in MS (128). In other autoimmune diseases, propionate also increases Treg cell numbers while reducing Th17-cell numbers, improving the prognosis of the disease (129–131). There are fewer clinical reports on RA, but in animal experiments conducted for RA, propionate reduction was found to completely reverse disease amelioration and suppress Treg cell increase and IL-10 production in mice with CIA. Exogenous propionate added to drinking water had protective effects in RA model mice, including reducing bone damage. Furthermore, propionate was found to have direct effects on T cells *in vitro* (132).

The differentiation of IL-10-producing regulatory B cells (Bregs) in response to gut microbiota-derived signals supports the maintenance of tolerance (133). A recent study found that the therapeutic effect of SCFAs on arthritis is also achieved by modulation of B-cell differentiation and is dependent on the FFA2 receptor. SCFAs reduced IgG, IgA, and IgE secretion, as well as plasma cell differentiation in human B cells, in a dose-dependent manner (134). Among SCFAs, butyrate levels were positively correlated with the frequency of IL-10-producing B cells (135). Compared to healthy controls, patients with RA and mice with arthritis have reduced levels of microbe-derived SCFAs. In mice, supplementation with the SCFA butyrate reduces the severity of arthritis, suppressing arthritis in a Breg-dependent manner (133). Taken together, the findings show that SCFAs may regulate immune balance through the Treg/IL-10/Th17 axis (131) or B cells (133).

### Effects of short-chain fatty acids on bone destruction

Gut microbes can act by inhibiting osteoclast proliferation and differentiation, inducing apoptosis, reducing bone resorption, or promoting osteoblast proliferation. In RA, synovitis damages the articular cartilage and the subchondral bone parenchyma and is an important cause of joint deformation and disability. One of the goals of recent research has been to address bone destruction to prevent joint deformities in patients with RA. SCFAs seem to positively affect bone composition in experimental arthritis models. Lucas et al. (136), using three independent experimental approaches, i.e., direct supplementation with SCFAs, feeding with a high-fibre diet (HFD), and bacterial transfer, found that mice subjected to direct supplementation with SCFAs and those fed an HFD exhibited increased systemic bone density, decreased bone resorption, and decreased osteoclast levels. They found that the ability of SCFAs to inhibit osteoclast differentiation and bone resorption was independent of the receptors gpr41 and gpr43. However, both butyrate and propionate induced metabolic switching to glycolysis in osteoclasts and significantly inhibited TRAF6, an important osteoclastogenic signalling component (137). Another study also found that treatment with SCFAs significantly attenuated the severity of inflammation in arthritis, with a systemic bone mass gain after treatment and significant downregulation of osteoclast-specific gene expression in bone, like TRAF6 and NFATc1 (136). Among SCFAs, butyrate inhibits osteoclast differentiation and reduces RA joint deformity (98). The mechanism may be related to reducate histone deacetylase, and changes of the KEGG pathways in osteoclast differentiation (98). SCFAs perform multiple immune and metabolic functions by binding to G protein-coupled receptors, relying mainly on the activation of free fatty acid receptor 2 (ffar2) (138). Recent studies have found that interfering with the gut microbiota using prebiotics may improve bone mass and alleviate skeletal problems. Tibial strength was positively correlated with increased levels of propionate and butyrate in the caecum. In addition, the results of different gut microbiota and micro-CT analyses suggest that the beneficial effects of prebiotics on bone mass may be related to the production of SCFAs in the caecum. SCFAs can promote phosphorus absorption, lower digestive pH, and enhance intestinal integrity, decreasing proinflammatory gene expression in the intestine and bone marrow and inhibiting osteoclastic bone resorption mediated by inflammatory cytokines (139). These findings highlight the importance of the gut–joint axis.

To date, a large number of studies have confirmed that RA can cause disturbance of the intestinal flora and SCFAs, while regulating the intestinal flora can restore SCFA levels, protect the intestinal barrier, regulate the immune balance, attenuate the inflammatory response in RA (129, 140), and inhibit bone destruction. These results illustrate that SCFAs play an essential role in the gut–joint axis.

## Role of tryptophan and its metabolites in the gut–joint axis

Tryptophan is an essential amino acid that cannot be synthesized by animal cells but has several physiological functions, and it plays an essential role in maintaining the balance between the intestinal microflora and intestinal mucosal immunity. In biology, tryptophan metabolism has been implicated in many of the most critical pathogenic conditions in humans. It is involved in several immune system diseases, including RA (141, 142), ankylosing spondylitis (143), osteoarthritis (144), Sjogren’s syndrome (145), MS (146, 147), and cancer (148). The indole pathway in the gut microbiota, the serotonin system in enterochromaffin cells, and the kynurenine pathway in immune cells and the gut wall are three metabolic routes for tryptophan metabolism in the gut. Tryptophan plays an important role in maintaining the intestinal barrier (149, 150), regulating immune balance (151), and alleviating bone destruction (141) through the above pathways.

### Tryptophan and its metabolites regulate intestinal barrier

The major metabolite of the essential amino acid tryptophan, such as indole-3-ethanol, indole-3-pyruvate, and indole-3-aldehyde, in the gut can prevent the increase in intestinal permeability under inflammatory stimuli by maintaining the integrity of the apical junctional complex and its associated actin regulatory proteins, including myosin IIA and ezrin, and these effects depend on the activation of the aryl hydrocarbon receptor (AHR) (150). In one study, tryptophan diet was found to alleviate the symptoms of dextran sulfate sodium (DSS)-induced enteritis, and with the increase of serum and colonic tryptamine levels, the levels of proinflammatory factors such as IL-6 were significantly decreased compared with those in the control group (152). Upon switching the carbon source from sugars to tryptophan, highly adapted lactobacilli, such as *Lactobacillus reuteri*, rapidly proliferate and produce indole-3-acetaldehyde, a ligand of AHR, which can promote AHR-dependent IL-22 transcription (153), and IL-22 initiates an il-18-dependent epithelial response loop by inducing phosphorylated STAT3 expression to reinforce the host intestinal defence (154). Meanwhile, IL-22 can also induce the secretion of antimicrobial proteins, promote colonization by diverse flora, inhibit colonization by *Candida albicans* and protect the mucosa from inflammatory destruction (146). Indole-3-formaldehyde (3-IALD), a tryptophan metabolite of the intestinal flora, plays a role in maintaining intestinal epithelial barrier integrity and suppressing inflammatory responses dependent on AHR/IL-22, which can affect the structure and function of the intestinal flora in mice, leading to an increase in the abundance of sugar-fermenting bacteria and SCFA-producing bacteria (155). Additionally, Lamas et al. (156). found that knockout of CARD9, a gene associated with antimicrobial sensitivity, impaired tryptophan metabolism in the gut microbiota, altered the IL-22 signalling pathway, and led to defective AhR activation, leading to decreased intestinal epithelial cell proliferation and increased apoptosis. After transplanting three strains of 3-IALD-metabolizing lactobacilli, AhR activation improved the intestinal inflammatory response and homeostasis. This illustrates that tryptophan metabolite has a crucial role in maintaining the intestinal barrier.

### Tryptophan and its metabolites regulate the immune balance

The Treg/Th17 cell balance plays an essential role in the progression of RA. One study found that the Treg/Th17 balance can be affected by modulating the gut microbiota composition and tryptophan metabolite levels (157). In a model of ankylosing spondylitis, indole-3-acetic acid, a metabolite of tryptophan, not only improved intestinal flora abundance and gut mucosal barrier function and suppressed proinflammatory cytokines but also upregulated the transcription factor forkhead box protein 3 (Foxp3) and increased Treg cell levels by activating the AhR pathway and downregulated the transcription factors retinoic acid receptor related orphan receptor γ t (ROR γ t) and signal transducer and activator of transcription 3 (STAT3) and reduced Th17 cell levels. In addition, oral tryptophan supplementation suppresses antigen-specific Th1 responses (158), which are vital in driving autoimmunity (159) and are closely correlated with RA severity. Clinical studies have found lower levels of tryptophan metabolites in the synovial fluid of RA patients compared to that in osteoarthritis patients (160) and reduced levels of ligands that activate AHR, leading to insufficient Treg cell generation and disruption of the Treg/Th17 balance; these findings indicate the importance of tryptophan supplementation for improving the Treg/Th17 balance in RA.

### Tryptophan and its metabolites alleviate bone destruction

Kynurenine, a tryptophan metabolite that accumulates with age, contributes to bone loss (161). Activation of the kynurenine pathway plays an important role during hMSC commitment to the osteoblast lineage *in vitro* (162). In the future, it may be an important target for prevention and treatment of bone destruction. In addition, a small fraction of tryptophan (1–2%) can produce serotonin in enterochromaffin cells *via* tryptophan hydroxylase 1 (TPH1), the expression of which is also regulated by SCFAs. The relevance of gut-derived serotonin in RA remains to be investigated, but serotonin may play a role in the control of bone remodelling, indicating the presence of a gut-bone axis (163, 164).

In conclusion, tryptophan metabolism plays a central role in the human body. Some recent evidence describes a complex cross-regulation involving the microbiota, diet, genetics, mucosal barrier, and immune function. However, as each of these factors is reciprocally affected, it is nearly impossible to delineate the hierarchy. Because genetic background, dysbiosis, and inflammation may alter this delicate balance, promoting an increase in specific tryptophan metabolites and a decrease in others and exacerbating or attenuating intestinal and systemic inflammation. So further research on the mechanisms of microbiota-host interactions is needed to refine targets and therapeutic interventions.

## Conclusions and perspectives

**Figure 1:**
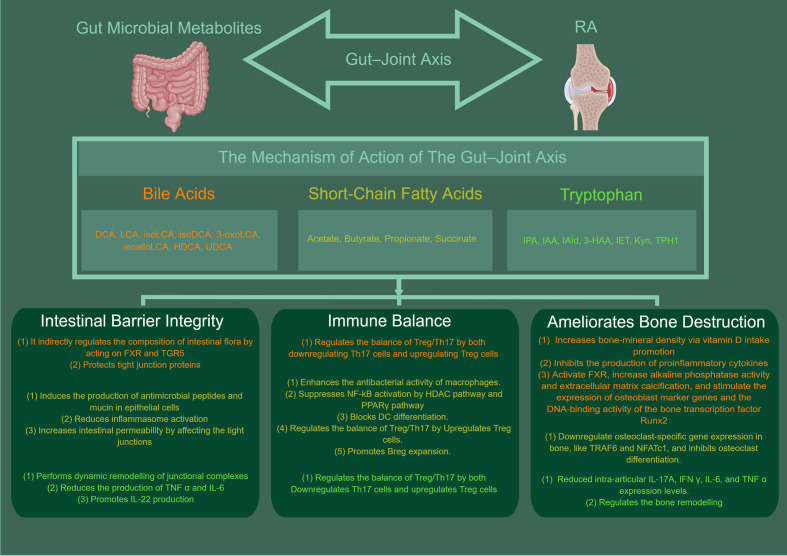
The mechanisms of gut microbial metabolites in RA. BAs, SCFA, TRP can protect the intestinal barrier by acting in a number of ways, such as directly *via* bactericidal effects or indirectly by acting on FXR and TGR5 to regulate the composition of the intestinal flora, modulate inflammatory factors (e.g., NF-κB, NLRP3, IL-6, IL-22), maintain the integrity of the apical linkage complex and its associated actin regulatory proteins and increase the levels of antimicrobial mucin peptides. In addition, it is apparent that intestinal microbial metabolites have a significant role in maintaining intestinal immune homeostasis. They can regulate the balance of Treg/Th17 or B cells, and enhances the antibacterial activity of macrophages. They can also suppresses NF-κB activation by HDAC pathway and PPARγ pathway and blocks DC differentiation. Meanwhile, the gut microbial metabolites can also be ameliorates bone destruction. The mechanism is related to increases bone-mineral density *via* vitamin D intake promotion, inhibits the production of proinflammatory cytokines, and ctivate FXR, increase alkaline phosphatase activity and extracellular matrix calcification. And they can stimulate the expression of osteoblast marker genes and the DNA-binding activity of the bone transcription factor Runx2, downregulate osteoclast-specific gene expression in bone, inhibits osteoclast differentiation. This picture was made by figdraw.

As with most acquired diseases in humans, the pathogenesis of RA needs to be fully understood to find mechanistically targeted therapies and achieve successful therapeutic responses. The complex gut microbiota is one of the factors contributing to RA. Gut microbial and metabolite alterations have been linked to the pathogenesis of RA and can provide therapeutic targets for the treatment of RA. Many studies have proven that the bacteria produce or modify major gut microbial metabolites (**Table 1**). They are associated with RA's intestinal barrier, systemic immunity, and bone metabolism (**Table 2**). The alterations in gut microbial metabolites are associated with the pathogenesis of RA (**Figure 1**). However, the mechanisms by which these changes are modulated require additional research in larger patient cohorts and animal models.. For example, the role of gut dysbiosis and gut barrier integrity has recently been investigated, and treatment with larazotide acetate (a recently developed zonulin receptor antagonist currently being evaluated for the treatment of celiac disease) in a mouse model of CIA restored gut barrier integrity and reduced joint inflammation. This has led to a desire to translate these benefits into clinical practice. However, few studies have explored their role in managing RA in human subjects. Studying these microbial metabolites could help us understand the gut–joint axis by identifying common active molecules produced by these bacteria, which could also be used as targets for new treatments for RA.

**Table 1:** Major gut microbial metabolites produced or modified by the bacteria.

Metabolites	Examples	Intestinal flora
BAs	DCA, LCA, isoLCA, isoDCA, 3-oxoLCA, isoalloLCA, HDCA, UDCA	Lactobacillus, Bifidobacterium, Clostridium, and bacteroid (38, 39)
SCFA	Acetate, Butyrate, Propionate, Succinate	Bifidobacterium, Lactobacillus (107), Bacteroides, Bacteroides fragilis (95), Faecalibacterium prausnitzii, Eubacterium rectale, Roseburia spp (94)
Trp	IPA, IAA, IAId, 3-HAA, IET, Kyn, TpH1	Ruminococcus, Escherichia coli, Peptostreptococcus russellii (153), Lactobacillus spp, Clostridium sporogenes (157)

BAs, bile acids; SCFA, Short-chain Fatty Acid; TRP, Tryptophane; DCA, Deoxycholic Acid; LCA, Lithocholic Acid; isoLCA, Isolithocholic acid; isoDCA, Isodeoxycholic Acid; 3-oxoLCA, 3-oxolithocholic acid; isoalloLCA, Isoallolithocholic acid; HDCA, hyodeoxycholic acid; UDCA, ursodeoxycholic acid; IPA, indole-3-pyruvate; IAA, indole-3-acetic acid; IAId indole-3-aldehyde; 3-HAA, 3-hydroxyanthranilic acid; IET, indole-3-ethanol; Kyn Kynurenine; TpH1, tryptophan hydroxylase 1.

**Table 2:** Mechanisms of gut microbial metabolites in RA.

Metabolites	Receptors	Functions	Mechanisms
BAs	FXR (33) TGR5 (61)	- Protects from intestinal barrier dysfunction	- Acts as an intestinal FXR and TGR5 agonist or antagonist to reduce proinflammatory cytokine production (80) - Protects tight junction proteins (104)
		- Maintains immune homeostasis	- Regulates the balance of Treg/Th17 by both downregulating Th17 cells and upregulating Treg cells (43, 44, 64, 65)
		- Ameliorates bone destruction	- Increases bone-mineral density *via* vitamin D intake promotion (69–71). - Inhibits the production of proinflammatory cytokines, such as TNF-α, IL-1β, and IL-6 (79) - Activate FXR, increase alkaline phosphatase activity and extracellular matrix calcification, and stimulate the expression of osteoblast marker genes BSP, OC, OPN and ALP and the DNA-binding activity of the bone transcription factor Runx2 (84)
SCFA	GPR41 (137) GPR43 (137) GPR109a (103)	- Protects from intestinal barrier dysfunction	- Induces the production of antimicrobial peptides and mucin in epithelial cells (108) - Reduces inflammasome activation (61) - Increases intestinal permeability by affecting the tight junctions (103, 104)
		- Maintains immune homeostasis	Enhances the antibacterial activity of macrophages (117) - Suppresses NF-κB activation by HDAC pathway and PPARγ pathway (113, 114) - Blocks DC differentiation (119) - Regulates the balance of Treg/Th17 or B cells (131, 133)
		- Ameliorates bone destruction	- Downregulate osteoclast-specific gene expression in bone, like TRAF6 and NFATc1, and inhibits osteoclast differentiation (136)
Trp	AhR (150)	- Protects from intestinal barrier dysfunction	- Performs dynamic remodelling of junctional complexes (150) - Reduces the production of TNF-α and IL-6 (150–152) - Increases IL22 expression (154)
		- Maintains immune homeostasis	- Regulates the balance of Treg/Th17 by both Downregulates Th17 cells and upregulates Treg cells (157)
		- Ameliorates bone destruction	- Reduced intra-articular IL-17A, IFN γ, IL-6, and TNF-α expression levels (126) -Regulates the bone remodelling (163, 164)

Bas, bile acids; SCFA, Short-chain Fatty Acid; TRP, Tryptophane; FXR, farnesoid X receptor; TGR5, G protein-coupled bile acid receptor 1; GPR, G protein-coupled receptor; AhR, Aryl Hydrocarbon Receptor; BSP, bone sialoprotein; OC, osteocalcin; OPN, osteopontin; ALP, alkaline phosphatase; TNF-α, Tumor necrosis factor-α; IL-1β, interleukin-1β; IL-6, interleukin-6; HDAC, histone deacetylase; PPARγ, peroxisome proliferator-activated receptor γ; DC, Dendritic cell; TRAF6, TNF receptor associated factor 6; NFATc1, Recombinant Nuclear Factor Of Activated T-Cells Cytoplasmic 1; IL22, interleukin-22; Th17, T helper 17; Treg, Regulatery T; IL-17A, interleukin-17A; IFN γ, interferon-γ.

## Author contributions

Conceptualization: BW, YL; Data curation: ZW, QC, XC,MD; Formal analysis: ZW, QC, YX, MD; Writing – original draft: XX, MW; Writing – review & editing: BW, YL. All authors contributed to the article and approved the submitted version.

## Funding

This work was supported by the Youth Fund of National Natural Science Foundation of China (no.: 82104848), the Natural Science Foundation of Chong Qing Postdoctoral Science Foundation (cstc2019jcyj-bshX0044), the Natural Science Foundation of Chongqing, China(cstc2020jcyj-msxmX0404), Chongqing Municipal Health Commission (Chongqing Traditional Chinese Medicine (2020) No. 20), Project of Chongqing Hospital of Traditional Chinese Medicine (cqzyymzygzs-001), and the “Xinglin scholar” hospital project of Chengdu University of traditional Chinese Medicine(YYZX2020056).

## Conflict of interest

The authors declare that the research was conducted in the absence of any commercial or financial relationships that could be construed as a potential conflict of interest.

## Publisher’s note

All claims expressed in this article are solely those of the authors and do not necessarily represent those of their affiliated organizations, or those of the publisher, the editors and the reviewers. Any product that may be evaluated in this article, or claim that may be made by its manufacturer, is not guaranteed or endorsed by the publisher.
